# Fracture in the Elderly Multidisciplinary Rehabilitation (FEMuR): study protocol for a phase II randomised feasibility study of a multidisciplinary rehabilitation package following hip fracture [ISRCTN22464643]

**DOI:** 10.1186/s40814-015-0008-0

**Published:** 2015-04-07

**Authors:** Nefyn H Williams, Claire Hawkes, Nafees Ud Din, Jessica L Roberts, Joanna M Charles, Val L Morrison, Zoe Hoare, Rhiannon T Edwards, Glynne Andrew, Swapna Alexander, Andrew B Lemmey, Bob Woods, Catherine Sackley, Pip Logan, David Hunnisett, Kevin Mawdesley, Clare Wilkinson

**Affiliations:** 1 Schools of Medical and Healthcare Sciences, Bangor University, Bangor, UK; 2 School of Psychology, Bangor University, Brigantia Building, Penrallt Road, Bangor, LL57 2AS UK; 3 Betsi Cadwaladr University Health Board, North Wales, UK; 4 School of Sports, Health and Exercise Science, Bangor University, George Building, Normal Site, Holyhead Road, Bangor, LL57 2PZ UK; 5 School of Health and Social Care Research, King’s College, Strand, London, WC2R 2LS UK; 6 School of Medicine, University of Nottingham, University Park, Nottingham, NG7 2RD UK; 7 North Wales Organisation for Randomised Trials in Health, Y Wern, Normal Site, Bangor University, Holyhead Road, Bangor, LL57 2PZ UK

**Keywords:** Proximal femoral fracture, Rehabilitation, Self-efficacy, Randomised controlled trial, Cohort, Focus group, Acceptability, Feasibility, Discrete choice experiment, Economic evaluation

## Abstract

**Background:**

Proximal femoral fracture is a common, major health problem in old age resulting in loss of functional independence and a high-cost burden on society, with estimated health and social care costs of £2.3 billion per year in the UK. Rehabilitation has the potential to maximise functional recovery and maintain independent living, but evidence of effectiveness is lacking. Usual rehabilitation care is delivered by a multi-disciplinary team in the hospital and in the community. An ‘enhanced rehabilitation’ intervention has been developed consisting of a workbook, goal-setting diary and extra therapy sessions, designed to improve self-efficacy and increase the amount and quality of the practice of physical exercise and activities of daily living.

**Methods/design:**

This paper describes the design of a phase II study comprising an anonymous cohort of all proximal femoral fracture patients admitted to the three acute hospitals in Betsi Cadwaladr University Health Board over a 6-month period with a randomised feasibility study comparing the enhanced rehabilitation intervention with usual care. These will assess the feasibility of a future definitive randomised controlled trial and concurrent economic evaluation in terms of recruitment, retention, outcome measure completion, compliance with the intervention and fidelity of delivery, health service use data, willingness to be randomised and effect size for a future sample size calculation. Focus groups will provide qualitative data to contribute to the assessment of the acceptability of the intervention amongst patients, carers and rehabilitation professionals and the feasibility of delivering the planned intervention. The primary outcome measure is function assessed by the Barthel Index. Secondary outcomes measure the ability to perform activities of daily living, anxiety and depression, potential mediators of outcomes such as hip pain, self-efficacy and fear of falling, health utility, health service use, objectively assessed physical function and adverse events. Participants’ preference for rehabilitation services will be assessed in a discrete choice experiment.

**Discussion:**

Phase II studies are an opportunity to not only assess the feasibility of trial methods but also to compare different methods of outcome measurement and novel methods of obtaining health service use data from routinely collected patient information.

**Trial registration:**

Current Controlled Trials ISRCTN22464643, UKCRN16677.

**Electronic supplementary material:**

The online version of this article (doi:10.1186/s40814-015-0008-0) contains supplementary material, which is available to authorized users.

## Background

Proximal femoral fracture, more commonly referred to as hip fracture, is a common, major health problem in old age [1], and as the population ages, the number of elderly people falling and fracturing their hips is projected to increase further. Occurrence of hip fracture is strongly associated with decreased bone mineral density, increased age, prior fragility fracture, low muscular strength levels, cognitive impairment, chronic disease, under-nutrition, frailty, poor physical functioning, vision problems and weight loss (especially muscle loss) [2]. Mortality is high with 25% dying within the following 12 months. A review of the long-term disability associated with proximal femoral fracture found that 29% of patients did not regain their pre-fracture level of functioning after 1 year in terms of restrictions of activities of daily living [3]. Many people who were living independently before their fracture lose their independence afterwards, so it imposes a large health and social care cost burden on society amounting to £2.3 billion a year in the UK, equating to approximately £6 million a day [4]. Tian et al. [5] explored Torbay’s (Devon) unique patient-level linked data set of National Health Service (NHS) and social care costs for older people in the 12 months before and after being admitted to hospital as a result of a fall. They found that the cost of hospital, community and social care cost services for each patient were almost four times as costly in the 12 months after admission, compared to the costs of the admission itself, and that the majority of costs occurred outside of the acute hospital setting. Particularly frail individuals may go onto have a further proximal femoral fracture resulting in additional disability and deaths [6].

Three phases of recovery from proximal femoral fracture have been described and are useful for research purposes [7]. The first phase is in the hospital, recovering from injury and surgery, and making safe to discharge. The second phase is rehabilitation either in an institution or at home. The final phase is the enduring stage where patients use their own previous health belief strategies to determine if and when they have recovered. The National Institute for Health and Care Excellence (NICE) has issued guidelines for the management of hip fracture [8]. As well as prompt surgical treatment, the guidelines recommend that the associated medical needs are assessed promptly by a physician specialised in caring for this patient group, who can also identify goals for a programme of multidisciplinary rehabilitation. Such rehabilitation starts whilst in hospital during post-operative recovery and continues in the community following hospital discharge. It is delivered by a multidisciplinary team depending upon patients’ individual needs at different times during their recovery and on the availability and accessibility of services in different areas. Rehabilitation has the potential to maximise recovery, enhance quality of life and maintain independence, but whilst individual components of such programmes show promise, there is insufficient evidence of overall effectiveness or cost-effectiveness or of processes of change and potential psychological mediators [9-13]. The aim of our study is to finalise a new rehabilitation intervention and to complete a feasibility study before a definitive randomised controlled trial (RCT). The design of the feasibility study is described here.

### Developing a rehabilitation intervention

We have completed the first phase of this research project to develop a new multidisciplinary rehabilitation intervention within the Medical Research Council’s (MRC) framework for complex intervention development [14]. This was informed by a realist review [15,16] of the rehabilitation literature, which differs from a traditional systematic review [17] in that it utilises a theory-driven approach and attempts to combine principles of conventional reviews as well as a philosophy of realism, thus adopting an explanatory rather than a judgemental approach to evidence synthesis [16]. It does not provide simple answers to complex problems but rather provides a flexible way of building an explanatory account of what works for whom under what circumstances, taking into account the heterogeneous nature of complex programmes or interventions [17,18]. Hence, we used this approach to determine the mechanisms behind multidisciplinary rehabilitation and to establish which components were effective (or not effective) for specific patient groups and in which circumstances [16]. In addition, we carried out a survey of current rehabilitation practice in the United Kingdom and focus groups of members of multidisciplinary teams and hip fracture patients to obtain their views about current rehabilitation programmes.

Mechanisms which the realist review highlighted as being important for successful outcomes included the following: tailoring the intervention to the patient; the regular practice of physical activities and activities of daily living; enhancement of psychological beliefs about recovery tasks, provision of motivation and a sense of ownership to engage in exercise; and co-ordinated provision of the multidisciplinary rehabilitation programme.

### Study objectives

The objectives of this study are the following:

1. To assess the acceptability of, and compliance with, the rehabilitation programme amongst patients, carers and clinicians and the fidelity of its delivery and to identify any adverse events.
2. To assess the feasibility of a future definitive RCT by assessing the number of eligible patients, monitoring recruitment and retention rates, exploring the willingness of patient participants to be randomised and the willingness of patients and carers to complete process and outcome measures.
3. To produce means and standard deviations of the quantitative measures so that effect sizes can be calculated for planning the future RCT.
4. To explore how the intervention creates change in self-efficacy and fear of falling as potential mediators for improving function.
5. To explore the methodological issues for conducting an economic evaluation alongside a future RCT including identifying the most efficient way of measuring patient level costs and health benefits, programme costs and potential payer stakeholders.
6. To explore the feasibility and quality of data on service use extracted from patient electronic records compared with patient reported outcome measures. If successful, replacing patient reported outcomes of service use with data collection by researchers and the NHS information technology (IT) staff for electronic records has potential to reduce participant burden in future studies (this is referred to as the triangulation study later in the paper).

## Methods/design

### Study design

**Figure 1:**
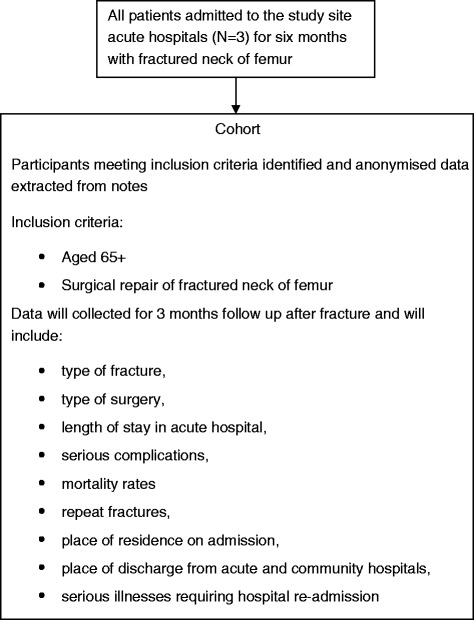
Cohort participant flow diagram.

**Figure 2:**
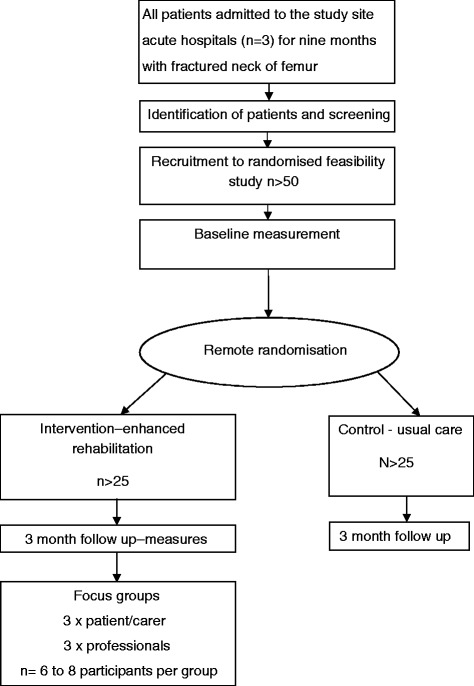
Randomised feasibility study participant flow diagram.

**Figure 3:**
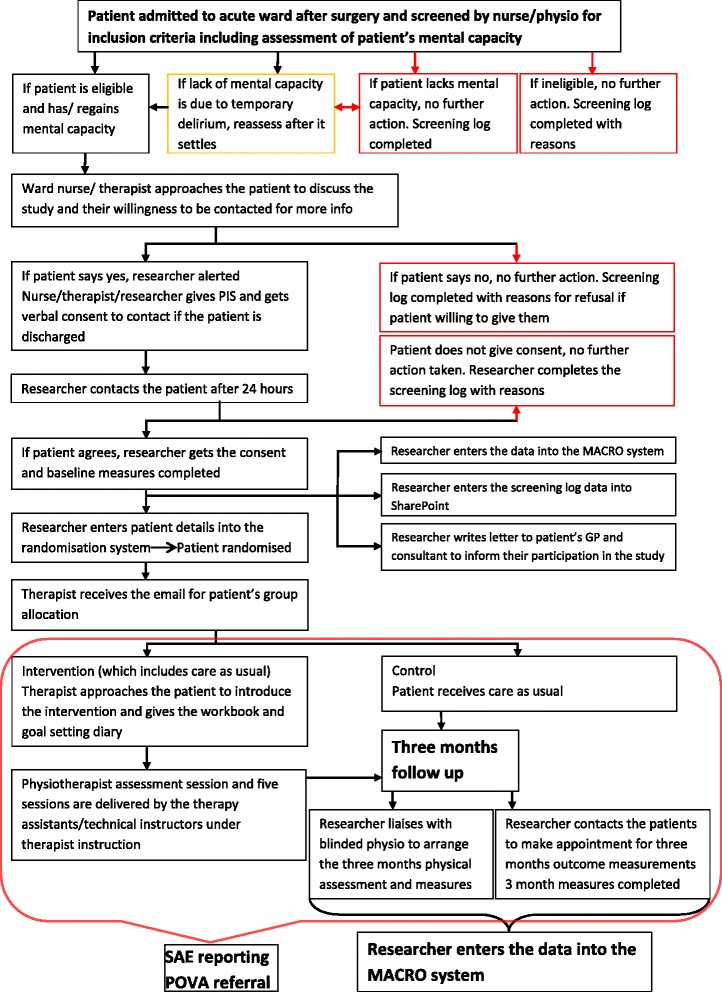
Randomised feasibility study recruitment process flow diagram.

Phase II comprises the second stage of the MRC framework for assessing complex interventions [14]. It will consist of an anonymous cohort study of all proximal femoral fracture patients (Figure 1) with an embedded randomised feasibility study (Figures 2 and 3) to achieve the objectives stated earlier. The acceptability and feasibility of the new rehabilitation programme will be assessed with further focus groups of the multi-disciplinary rehabilitation teams, hip fracture patients and their carers. The role of behavioural cognitions will be examined as process measures. It will also contain a sub-study to explore the potential for collecting health resource use from routinely collected electronic patient records.

### Cohort study

#### Selection of subjects for cohort study

The cohort will consist of an anonymised data set of all patients 65 years of age and over admitted to the three main acute hospitals of Betsi Cadwaladr University Health Board (BCUHB) in North Wales (Wrexham Maelor, Ysbyty Glan Clwyd and Ysbyty Gwynedd) with hip fracture during the first 6 months of the study period; who will subsequently be followed up for 3 months. BCUHB is the largest health board in Wales with a population of 692,000. Data collected will include type of fracture, type of surgery, length of stay in acute hospital, serious complications, mortality rates, repeat fractures, place of residence on admission, place of discharge from acute and community hospitals and serious illnesses requiring hospital re-admission. Audit figures returned to the National Hip Fracture Database give a conservative estimate that approximately 100 to 180 patients are admitted to each of these hospitals (a total of 300 to 540) in a 6-month period [19].

#### Cohort study outcomes

From the cohort anonymised data set, we will record the following:

- The number of patients aged over 65 years admitted with a proximal femoral fracture.
- The number of patients who fulfil the inclusion criteria for the randomised feasibility study.
- The number of deaths, serious complications such as falls and repeat fractures, serious illness requiring hospital re-admission and discharged to institutional care. This will include details such as the type of ward and the type of residential care in order to calculate the cost per night locally. This will be compared with the Department of Health reference costs for proximal femoral fracture related hospital stays.

### Randomised feasibility study

#### Selection of subjects for randomised feasibility study

Of all those admitted, we anticipate that we will be able to identify and invite 150 patients (50% of a conservative estimate of the total) from the three hospital sites across BCUHB during the 6-month study period to participate in the embedded feasibility study comparing usual care with the enhanced rehabilitation package using a randomised design. We will attempt to recruit 50 patients to this randomised feasibility study. The number of eligible patients, the recruitment and retention rates and the number who completed the outcome questionnaires will be recorded. It will be important to determine whether random allocation to either intervention arm is acceptable to patients, carers and clinicians providing the service. The feasibility study will also be an opportunity to test a package of process and outcome measures, including economic measures, for the main trial and to inform the effect size for a future sample size calculation. These patients will also be recruited to a triangulation study which aims to compare the quality of data collected about service use from patient-reported outcome measures compared with extracting the same information from patient electronic records.

#### Inclusion criteria for patient participants to feasibility study

We will aim to recruit older adults recovering on an orthopaedic ward with proximal femoral fracture who were previously living independently and who have recently received surgical treatment. The specific inclusion criteria are as follows:

- Age 65 years or older;
- Recent proximal hip fracture including the following types of fracture: intracapsular, extracapsular (pertrochanteric, intertrochanteric, reverse oblique or subtrochanteric);
- Surgical repair by replacement arthroplasty or internal fixation;
- Recovering as an in-patient on an orthopaedic ward, transferred to an in-patient rehabilitation ward or who have been discharged home;
- Living in their own home prior to hip fracture;
- Capacity to give informed consent; and
- Living and receiving rehabilitation from the NHS in the area covered by BCUHB.

#### Exclusion criteria

The exclusion criteria are as follows:

- Living in residential or nursing homes prior to hip fracture; and
- Participants who are not able to understand Welsh or English.

#### Informed consent - patient participants in the trial

Clinical staff on the orthopaedic wards of the three main hospitals in BCUHB will screen patients for eligibility. If eligible, the clinicians will approach the potential participant to see if they would be interested in taking part and willing to be seen by a researcher. The study team researchers supported by National Institute for Social care and Health Research (NISCHR) Clinical Research Centre (CRC) research professionals will then recruit patients following the study’s informed consent process which has been reviewed and approved by the NHS research ethics committee (see Additional files 1 and 2).

It is possible that during the study some participants’ capacity may change. Consequently, at follow-up, the researcher will be asked to assess whether the participant no longer has capacity to give informed consent when arranging the follow-up visit. If the patient no longer has capacity, no follow-up data will be collected, but the baseline and any other data collected to this point will be used in the analysis.

The study is taking place in an area where there are two official national languages, Welsh and English. Consequently, participants will be given a choice of Welsh or English language patient information sheets and informed consent forms. Where validated outcome measures exist in the Welsh language, participants will have the option to complete them in that language.

The researcher will complete the outcome measures with the patient participant before randomisation. The patients’ general practitioner and treating consultants will be informed of their participation and a record made in their medical records.

#### Informed consent - carer participants

Carers for the purpose of this study are defined as people caring for a hip fracture patient recruited to the study by providing them with face to face support most days in a week including help with activities of daily living and/or physical care. They may be a relative or a friend. The study team researchers supported by the NISCHR CRC research professionals will identify and recruit carers following the study’s informed consent process (similar to Additional files 1 and 2). Carers will be asked to complete a carer burden questionnaire [20] at baseline and at follow-up.

#### Randomisation

Patient participants who give their informed consent will complete baseline process and outcome measures before being individually randomised. The randomisation will be performed by dynamic allocation [21] to protect against subversion whilst ensuring that the trial maintains good balance to the allocation ratio of 1:1 both within each stratification variable and across the trial. Participants will be stratified by: (1) hospital and (2) gender.

Randomisation will be requested by the researcher who has taken informed consent and will be achieved by secure web access to the remote randomisation centre at the North Wales Organisation for Randomisation Trials in Health (NWORTH), Bangor University. This system will be set up, maintained and monitored independently of the trial statistician or other trial staff. The randomisation procedures will be aligned with NWORTH standard operating procedure 5.01 to ensure best practice. The key to the randomisation code will be held centrally by NWORTH.

#### Blinding

Collection of outcome measures, including physical function measures, and data analysis will be performed blind to treatment allocation. This is a pragmatic study comparing two rehabilitation interventions, so it will not be possible to blind participants or their clinicians to treatment group allocation.

#### Withdrawal of participants

Participant withdrawal from the study will not affect their medical or social care, and this point will be emphasised in the patient information sheet and during the informed consent process. Similarly, withdrawal of carer participants will not affect the medical or social care of the hip fracture patient they are caring for.

Non-completion of the follow-up questionnaires or physical function tests will not constitute formal withdrawal from the trial, and unless the participant requests withdrawal of their data completely, it may be used to impute values for the analysis. The imputation of missing values will ensure that the dataset is utilised to its full power.

#### Expected duration of feasibility study

Participants will be recruited over a 9-month period and followed-up for 3 months.

#### Interventions

An ‘enhanced rehabilitation’ intervention will be compared with usual rehabilitation care. Usual care consists of a multi-disciplinary rehabilitation delivered by the acute hospital, community hospital and community services depending on patients’ individual needs at different times during their recovery and on the availability and accessibility of services in different areas. The multidisciplinary team delivering care and rehabilitation includes orthopaedic surgeons, orthogeriatricians, nurses, physiotherapists, occupational therapists, dieticians, pharmacists, general practitioners (GPs) and social workers. The settings for care include acute orthopaedic or orthogeriatric wards, rehabilitation units in community hospitals, rehabilitation beds in care homes, the patient’s own home and care home settings all delivered by a variety of community teams in both health and social care services.

The main aim of the intervention is to enhance usual rehabilitation by increasing the amount and quality of patients’ practice of physical exercise and activities of daily living in order to improve their functional outcomes at the 3-month follow up. We also hypothesise that improving patients’ self-efficacy will increase their motivation to engage in the rehabilitation process, improve the quality and quantity of this practice and increase their engagement.

We will endeavour to enhance rehabilitation self-efficacy [22] by means of a patient-held information workbook and goal-setting diary given to the participant in the acute hospital which they will keep throughout the follow-up period of the study. Six additional therapist/technical instructor sessions will be available to patients once they return home or are admitted permanently to a care home. These extra sessions will be tailored to individuals’ needs at the discretion of the community occupational therapist or physiotherapist responsible for their care in liaison with the therapists allocated to deliver the extra sessions.

The objectives of the workbook and goal-setting diary are as follows:

1. To give patients better understanding of what has happened physically to them and broadly what to expect during their recovery;
2. To provide information and contact details on rehabilitation services that may be available to them as they progress in their rehabilitation (e.g. intermediate care teams, social services enablement teams, outpatient physiotherapy, falls prevention groups, national exercise referral services);
3. To enable them to work collaboratively with their therapist to set realistic goals and monitor progress of their rehabilitation in order to improve the quality and the quantity of the physical and activities of daily living exercises they are given;
4. To improve patients’ self-efficacy by the following:
  - Encouraging the patient to set mobility and activities of daily living goals that they want to achieve and to discuss what might facilitate or impede their attainment with their therapist.
  - Monitoring these goals by keeping a diary of progress made, which will provide feedback in the form of self-reflection and reflection with the therapist, highlighting success and mastery that is known to be important for improving self-efficacy [23].
5. To improve communication between hospital and community services and between the patient and all the different professionals and services they come into contact with during their rehabilitation;
6. To reduce patients’ fear of falling by improving self-efficacy for avoiding falls and exercising; and
7. To signpost patients to local follow-on community programmes such as exercise referral and falls prevention services with contact details.

**Figure 4:**
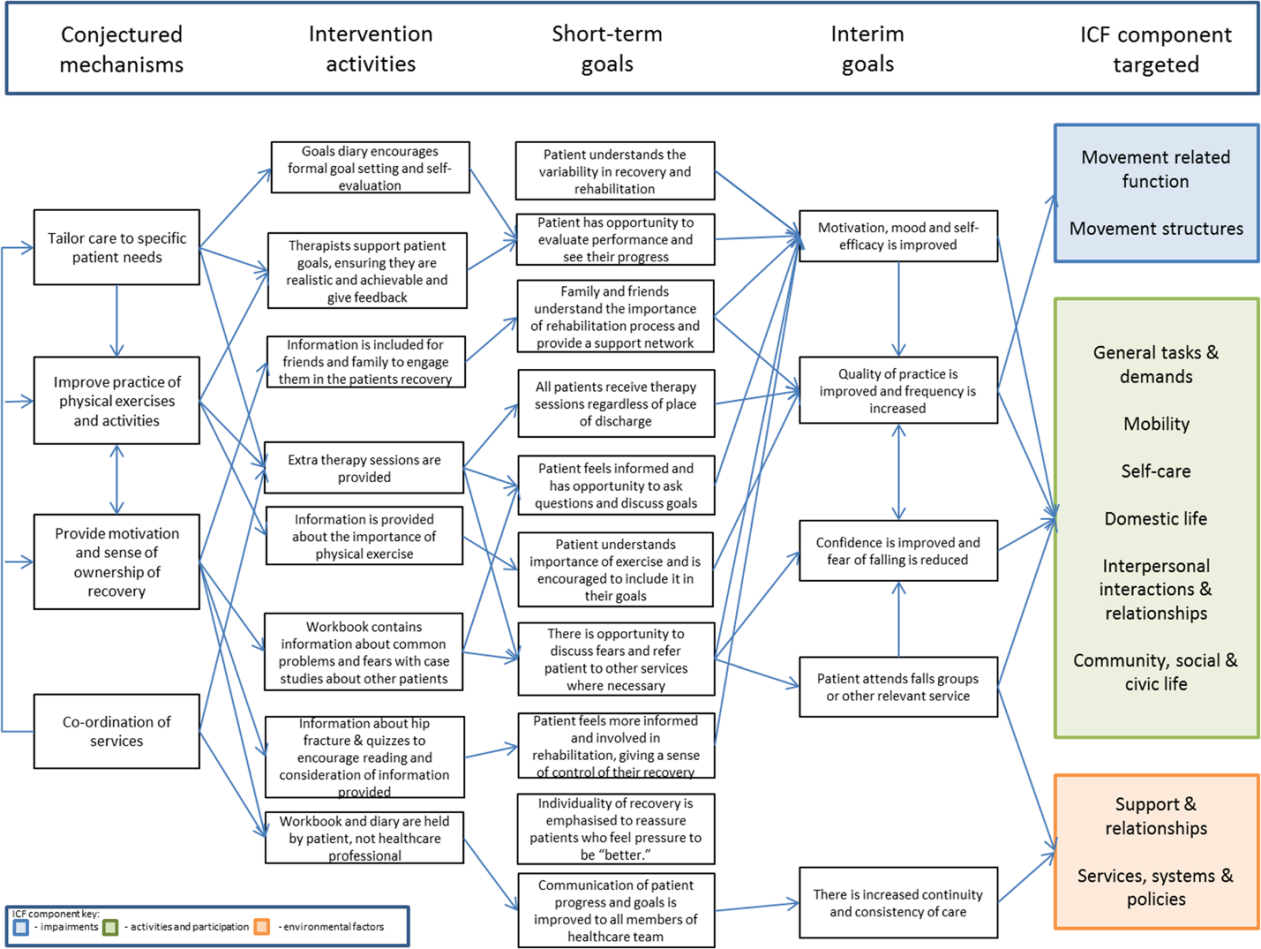
Logic model of enhanced rehabilitation intervention following proximal femoral fracture.

**Figure 5:**
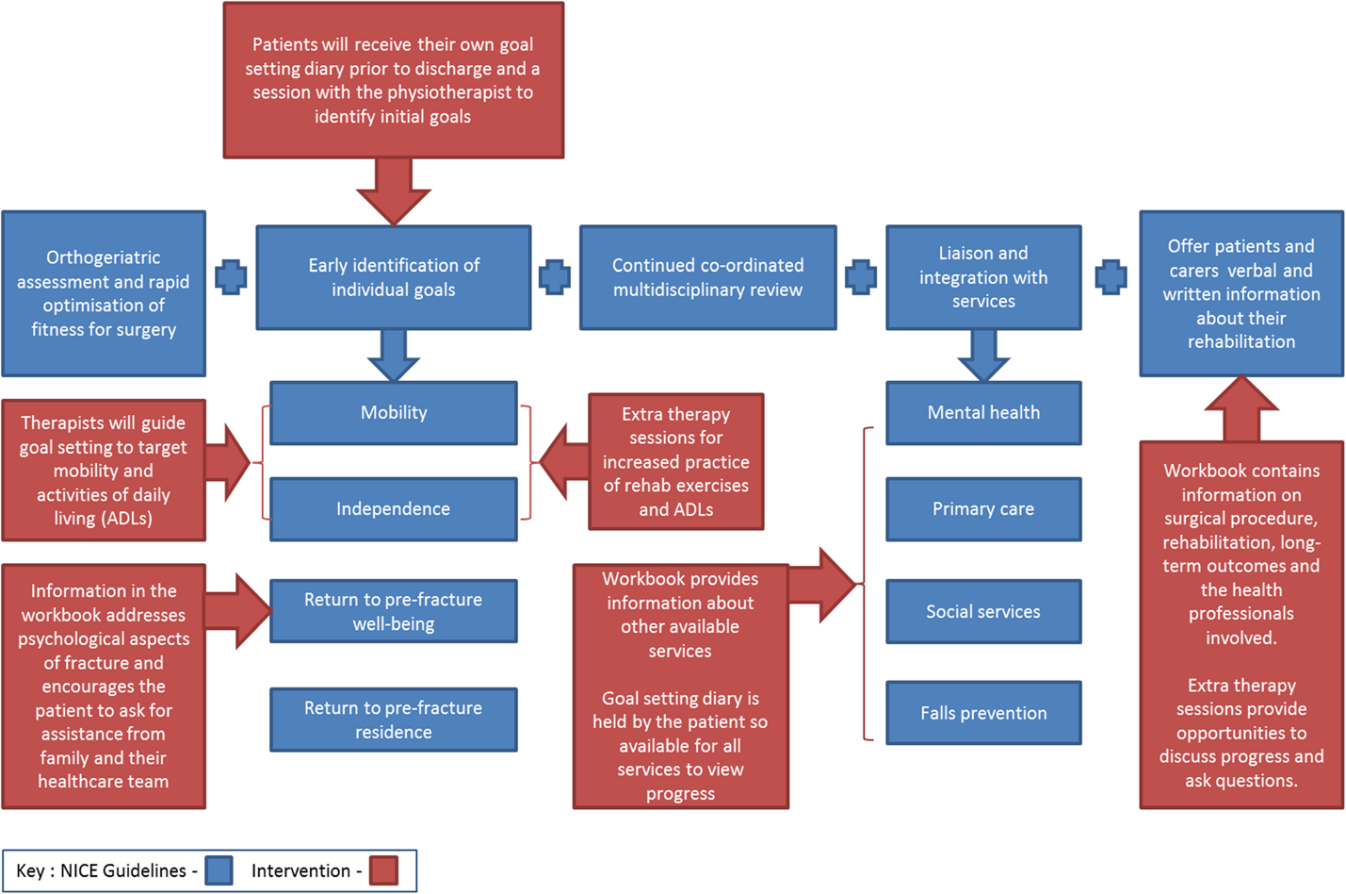
Mapping the rehabilitation intervention to the NICE recommendations for the management of hip fracture.

A logic model has been developed which describes how the study team has linked the programme theories from the realist review with the components of the intervention, the short-and long-term goals of the intervention, and functional outcomes in terms of the International Classification of Functioning, Disability and Health (ICF) [24] (Figure 4). We have also mapped the intervention components to the NICE recommendations for the multidisciplinary rehabilitation of hip fracture [8] (Figure 5). Therapists in BCUHB have contributed to the development and finalisation of the workbook, in particular, commenting on usability, accuracy and local information issues.

#### Delivery of the intervention

The intervention will be delivered by the therapists and their assistant staff employed by BCUHB. The therapy service managers in BCUHB have agreed to organise the extra sessions for patients in the intervention arm of the study, which will be funded by NHS excess treatment costs. The extra six sessions of rehabilitation will be delivered by additional physiotherapists and therapy assistants. This will be done in collaboration with the existing therapists delivering the usual care. The intervention therapy teams will be alerted to which group the patient is randomised and will arrange a time for an initial assessment and goal-setting session to visit the patient in the acute hospital or in a community setting. The remaining five sessions will be used according to individual’s need and the individual’s rehabilitation plan.

#### Randomised feasibility study outcomes

The outcomes will be collected in a variety of ways. Demographic data will be collected from patients and their records. Recruitment rates will be collected by researchers from their screening and recruitment records. At baseline and 3 months follow-up, patient-completed outcome measures will be completed by participants, assisted by NISCHR CRC professionals or a member of the research team who will be blind to treatment allocation. Participants will also be given the choice to complete validated versions in Welsh where they exist. At baseline, fewer patient-completed outcome measures will be used than at 3-month follow-up as we wish to reduce the burden on patients at a time shortly after surgery. Physical function will be objectively assessed by the researcher at baseline using the grip strength test. At 3 months follow up, a physiotherapist will measure other objective tests of physical function, including the grip strength test. These will be performed in the physiotherapy gym or if the patient is unable to travel in their own home. The timing of outcome assessments is summarised in Table 1.

**Table 1:** FEMuR protocol schedule of forms and procedures

Event	Baseline	Timeline post randomisation		
		During 3 months	At 3 months	Post 3-month follow-up
Eligibility screening and consent for randomised feasibility study				
Patient	X			
Carer	X			
Outcome measurement for feasibility study				
Cognitive status				
•AMTS [25]	X			
Primary				
•Barthel index [27]	X		X	
Secondary				
•NEADL Scale [28]	X		X	
•HADS [29]	X		X	
Process				
•VAS for hip pain [30]	X		X	
•GSES [32]	X		X	
•FES-I [33,34]			X	
•SES [35]			X	
•VAS-FoF [36]			X	
Health economic				
•EQ-5D 3L [37]	X		X	
•ICECAP-O [38,39]	X		X	
•CSRI [40]	X		X	
•DCE [43]			X	
Physical				
•Grip strength [45]	X		X	
•30-s sit-to-stand [46,47]			X	
•8-ft (2.5 m) get-up-and-go [47]			X	
•50-ft (15.4 m) walk [48]			X	
Carer strain index	X		X	
Therapist process outcomes and use of the intervention workbook				
•Date of extra session		X		
•Whether the session is face to face or indirect		X		
•Where the face to face session is held		X		
•If the session is face to face, time is spent on assessment, exercise, ADL practice, working on the workbook etc.		X		
Qualitative follow up patients and carers focus groups/individual interviews - (invited)				
Patients			X	X
Carers			X	X
Recruitment and consent of staff to focus groups		X	X	X

#### Routinely collected demographic, clinical and recruitment data

During recruitment to the feasibility study, we will collect information on the number of patients approached, the number eligible, the number who did and did not consent, and where possible the reasons why. The number of eligible patients who fulfilled the inclusion criteria and were willing to be randomised will be expressed as a percentage of the numbers in the cohort dataset. In addition, we will record the number who withdraw after baseline assessment and randomisation, and the number who complete the various outcome measures at baseline and 3 months follow-up. The researchers who administer the outcome measures will record the reasons for any non-completion.

The following demographic data and descriptors will be collected:

- Date of birth (age)
- Gender
- Type of fracture
- Type of surgery
- Living arrangements
- Place of residence prior to admission
- Place of discharge from acute and/or community hospital

#### Cognitive status

- Abbreviated Mental Test Score (AMTS) [25].

The AMTS is a test with evidence of validity that is widely used in clinical and research settings in the UK for detecting and monitoring cognitive impairment. This will be used as a baseline description of the level of cognition. It is brief (ten items) and recommended for cognitive screening in acute settings in the Alzheimer’s Society (2013) tool-kit [26] ‘Helping you to assess cognition: a practical toolkit for clinicians’. It is generally considered to be easily administered and well tolerated by raters and subjects. The score range is 0 to 10 with higher scores indicating worse cognitive function.

#### Patient completed measures - primary outcome

- Barthel index [27].

This is a patient- or assessor-completed outcome measure of current functional status measuring individuals’ ability to care for themselves. It has evidence of validity when used in patients with musculoskeletal or neuromuscular disorders and is considered easy to use, reliable and sensitive to change. It focuses on the person’s level of independence on the following items: feeding, bathing, grooming, dressing, bowel function, bladder function, toilet use, transfers, mobility on level surfaces and stairs. It will be measured at baseline and at the 3-month follow-up assessment. The score range is 0 to 20 with lower scores indicating increased disability.

#### Patient completed measures - secondary outcomes

- Nottingham Extended Activities of Daily Living (NEADL) Scale [28].

This is a patient-completed outcome measure of activities of daily living from the previous 4 weeks which has evidence of validity in stroke patients. The NEADL is a record of actual activity rather than capability, scoring patients in the areas of mobility, kitchen, domestic and leisure activities. A higher score indicates a greater level of independence. When assessed at baseline, it will assess the participant’s functional capacity prior to hip fracture. It will also be used at the 3-month follow-up assessment to assess the degree of functional recovery. The score range is 0 to 66 with higher scores indicating greater independence.

- Hospital Anxiety and Depression Scale (HADS) [29].

This is a patient-completed outcome measure of anxiety and depression. It is designed to measure anxiety and depression in patients with physical health problems. It has seven items related to common symptoms of anxiety and seven for depression. Patients are asked whether they experience the symptom definitely, sometimes, not much or not at all. The HADS was designed for use in the hospital setting but has been used successfully with the general population. This measure will be used at baseline and at the 3-month follow-up assessment. The two sub-scales have a range of 0 to 21 with higher scores indicating increased anxiety or depression.

#### Process measures (potential mediators of outcomes)

- Visual Analogue Scale (VAS) for hip pain intensity [30].

This is a patient-completed visual analogue scale of current hip pain intensity. Hip pain following surgery is an important factor affecting rehabilitation and will be measured at baseline and at the 3-month follow-up assessment. We have chosen a VAS as there is evidence of validity compared with the Oxford Hip Score [31] whilst being simpler and quicker to complete, thus reducing the burden on patients. The range is 0 to 10 on a segmented line.

- General Self-Efficacy Scale (GSES) [32].

The GSES is not behaviour specific and is chosen as a measure of general confidence when facing challenge. In order to assess change over time in such expectancy-based cognitions pre and post intervention (as well as test between group differences at follow-up comparing intervention with control), we have chosen this short self-efficacy scale for baseline. It has evidence of validity in the populations of older people and surgical patients. The range is 10 to 40 with higher scores indicating increased self-efficacy. The measure will also be completed at 3-month follow-up with the more behaviour specific Falls Efficacy Scale - International and the self-efficacy for exercise scale (see below).

- Falls Efficacy Scale - International (FES-I) (self efficacy) [33,34].

The FES-I measures how concerned a patient is about falling when performing activities of daily living both inside and outside of the home. The scale details 16 activities which the patient must rate from 1 (not at all concerned) to 4 (very concerned) with regards to how concerned they would be about falling if they performed the activity. The range is 16 to 64 with higher scores indicating a greater fear of falling. The FES-I has been used successfully in older patients both without and with mild cognitive impairment.

- Self-efficacy for exercise scale [35].

The self-efficacy for exercise scale is a revision of unpublished self-efficacy barriers to exercise measure. The scale consists of statements regarding the participant’s confidence that they could exercise for 20 min, three times a week, depending on factors such as pain and mood. The participants are instructed to use numbers from 0 (not confident) to 10 (very confident) to rate their expectations. This measure assesses the participant’s present expectations and so will be used only at the 3-month follow-up as pain from surgery would likely be the major factor in these expectations at baseline and would not measure normal levels of self-efficacy for the patient. The range is 0 to 90 with higher scores reflecting more confidence in capability.

- Visual Analogue Score - Fear of Falling (VAS-FoF) [36].

This is a patient-completed visual analogue scale for fear of falling. A VAS is useful as it is easy to administer and brief. The range is 0 to 10 on a segmented line with higher scores indicating greater fear of falling. It has previously been used in older adults with and without cognitive impairment with good results [25] and will be used to measure fear of falling in our study at 3 months follow-up.

#### Health economic measures

- EuroQol EQ-5D (three levels) [37].

This is a patient-completed index of health-related quality of life, which gives a weight to different health states. It consists of five dimensions: mobility, self-care, usual activities, pain/discomfort, and anxiety/depression. Each has three possible responses. The responses record three levels of severity (no problems, some/moderate problems and extreme problems) which is then converted to a health utility weight using UK norms. It will be used at baseline and at the 3-month follow-up assessment and allows the calculation of quality-adjusted life years (QALYs), using area under the curve method which will be used as part of the economic analysis.

- ICEpop CAPability measure for Older people [38,39].

This is a patient-completed measure of capability in older people that focuses on well-being rather than health. It comprises five attributes: attachment (love and friendship); security (thinking about the future without concern); role (doing things that make you feel valued); enjoyment (enjoyment and pleasure); and control (independence). Each attribute has four possible responses. The responses record the extent of capability (all, a lot, a little, and none), and the five-digit outcome is converted to capability utility derived from UK norms. It will be used at baseline and at the 3-month follow-up assessment as part of the economic analysis.

- Client service receipt inventory (CSRI) [40].

The CSRI is a questionnaire for collecting retrospective information about study participants’ use of health and social care services, including voluntary services (e.g. charity services), including the components of the rehabilitation programme. This information will be combined with national sources of reference unit costs [41,42] in order to calculate health and social care service costs for the economic evaluation. It will be used at baseline and at the 3-month follow-up assessment as part of the economic analysis.

- Discrete choice experiment [43,44].

A discrete choice experiment (DCE) [43,44] will explore participants’ preferences for rehabilitation services. Hypothetical rehabilitation services (A and B) will be presented to each participant according to a set number of characteristics, each of which has different levels. These characteristics and levels will create a set of alternative scenarios. In each scenario, the participant will be asked which service they prefer by ticking ‘A’ or ‘B’. The characteristics and levels will be chosen for the DCE based upon the outcomes of the literature review and focus groups conducted in phase I of the study. It will be used at the 3-month follow-up assessment as part of the economic analysis.

#### Objective measures of physical function

- Grip strength [45].

This is an objective measure of physical function that will be administered by the researcher administering the patient-completed questionnaires. Grip strength correlates well with general fitness and muscle strength relating to physical function. It is also a more appropriate measure for use at baseline, as performing other physical assessments may carry risk to patients at this time point or would be likely to primarily reflect post-operative pain and not overall function. Grip strength will be measured at baseline and at the 3-month follow-up assessment. Other objective measures will be administered by a physiotherapist at the 3-month follow-up and are listed below:

- Thirty-second sit-to-stand [46,47].

From a seated position in a chair with no armrests, the participant rises to a full stand and returns to a fully seated position without using their arms to support themselves. An observer measures the number of stands completed in 30 s. The 30-s sit-to-stand is used to measure lower body strength and is useful in older adults because it is part of everyday activities, e.g. getting off the toilet and getting in and out of a car and a chair. It correlates reasonably well with other measures of lower body strength such as knee extensor and knee flexor strength and has been shown to have good test-re-test reliability in older adults living in a community setting [46,47].

- Eight-foot (2.5 m) get-up-and-go test [47].

Timed up-and-go test (also known as ‘8 get-up-and-go’) is used to assess mobility, agility and balance. An observer measures the time taken for a participant to stand up from a chair, walk 8 ft (2.5 m) with or without a walking aid, turn 180°, walk back to the chair and sit down. There is evidence of validity and reliability [33-36].

- Fifty-feet (15.4 m) walk test [48].

The participant is brought to start on a level 50-ft (15.4 m) walk test course (25 ft out and 25 ft back) and is asked on the command ‘go’ to walk as quickly as possible to the 25-ft (7.7 m) mark and back. An observer records the amount of time taken from the command ‘go’ until the starting line is crossed on the way back. It has been shown that there are correlations between the recorded gait time and muscle strength and also with the ability of older people living in the community to carry out activities of daily living [49].

#### Carer completed measure - secondary outcome

- Caregiver strain index [18].

Carers who have been recruited onto the study will be asked to complete this measure. It is a 13-item tool that measures strain related to care provision. There is at least one item for each of the following major domains: employment, financial, physical, social and time. Positive responses to seven or more items indicate a greater level of strain. It can be used to assess individuals of any age who have assumed the role of caregiver for an older adult. It will be completed at baseline and at the 3 months follow-up. The range is 0 to 13 with a higher score indicating greater strain.

#### Therapist process outcomes and use of the intervention workbook

In order to describe the rehabilitation programme in both arms of the feasibility study, we will access routinely collected data that therapists complete on their ‘Therapy Manager System’. An information technology manager at BCUHB will extract the following data and return them anonymously to the research team, identifiable only by participants’ study ID. The intervention therapy teams will complete a paper record of how they use the extra sessions which also form part of patients’ clinical records. A BCUHB member of staff will extract the following from these records and return it to the research team:

- Patient study identification, date of extra session;
- Whether the session is face to face or indirect;
- Where the face-to-face session is held; and
- If the session is face to face, how much time is spent on different aspects such as assessment, exercise, activity of daily living (ADL) practice and working on the workbook.

We will assess whether the fidelity and dose of the enhanced rehabilitation programme delivered to participants is consistent with our programme theory. We will describe how the programme is delivered along with patients’ views and their use of the workbook which will be collected through focus groups described later. The workbook contains a page of questions and Likert scale type response options to encourage participants to provide feedback on their workbook. Researchers will also collect the diary sections to assess how they are used. We will evaluate engagement with the workbook by counting how many diaries are used, how regularly they are filled out, and whether goals are set and quizzes completed.

#### Triangulation study of service use information

The health service use data obtained from the patient completed CSRI questionnaires will be compared with the same information obtained from routinely collected data recorded on computerised patient records. The data will be collected by NWORTH and BCUHB IT staff.

### Assessment of safety

#### Recording adverse events

All adverse events will be recorded in this study. There are adverse events (AEs) and serious adverse events (SAEs). Adverse events will include the following:

- Non-injurious falls;
- An exacerbation of a pre-existing illness;
- An increase in frequency or intensity of a pre-existing episodic condition;
- A condition (even though it may have been present prior to the start of the feasibility study) detected after the start of the study; and
- Continuous persistent disease or symptoms present at baseline that worsens during the study.

The following will not be included as adverse events:

- Medical or surgical procedures where the condition which leads to the procedure is the adverse event;
- Pre-existing disease or conditions present before treatment that do not worsen; and
- Overdose of medication without signs or symptoms.

SAE will be any medical event that:

- Results in death;
- Is life-threatening (refers to an event during which the participant was at risk of death at the time of the event; it does not refer to an event which might have caused death had it been more severe in nature);
- Falls and repeat fractures;
- Requires hospitalisation or prolongation of existing hospitalisation;
- Results in persistent/significant disability or incapacity; and
- Other important medical events that, based upon appropriate medical judgement, may jeopardise the participant and may require medical or surgical intervention.

#### Process for recording adverse events

All adverse events will be recorded by researchers when they are made aware of the event by the patient, carer, the treating clinicians, or therapists. Adverse event reporting information will be included in the training given to the therapy teams delivering the intervention and they will be given copies of the adverse event reporting forms (Additional file 3) and details of how to return them to the research team. Details of the adverse event reporting procedure will also be included in letters to the participants’ GP and consultant informing them of their participation in the study. The adverse event form will have two sections, the first is for the healthcare professional to complete and return to the study manager. The study manager will liaise with the chief investigator who will determine whether the adverse event is serious or not and whether it is related to the study. The chief investigator will complete the second part of the form. All serious adverse events, along with the chief investigator’s assessment of whether it is related to the study, will be sent to the Data Monitoring and Ethics Committee (DMEC) for a second opinion. The trial study manager will record the information on the study master file and inform the clinical trials unit manager. Study-related serious adverse events will be reported to the sponsor and to the academic school (Schools of Medical and Healthcare Sciences, College of Health and Behavioural Science) within 24 h of being determined as serious. They will also be reported to the DMEC chair and the research ethics committee.

#### Referral of vulnerable adults to protection agencies (protection of vulnerable adults referral)

Staff and researchers recruiting patients have been provided with statutory protection of vulnerable adults (POVA) training by the BCUHB within vulnerable adult protection framework [50] in accordance with Welsh Government guidance [51,52]. A mechanism of immediate risk assessment and onward referral to appropriate local authorities, police and BCUHB POVA hub has been developed within the framework of the Human Rights Act 1998 [53] and Data Protection Act 1998 [54] if abuse or neglect is suspected, observed or disclosed by the participants.

#### Statistics

##### Sample size

We estimate that we will recruit 25% of eligible patients with a proximal femoral fracture admitted to the three acute hospitals in BCUHB and randomise them to either the enhanced rehabilitation programme or usual care. In order to estimate the standard deviation of the primary outcome measure (Barthel index), which will be used in a power calculation for a future definitive RCT, with a high level of confidence a sample size of at least 50 participants completing the trial is advisable [55].

#### Statistical analysis

The main outcomes of the feasibility study will be the descriptive statistics of recruitment and retention figures as follows:

- The number of patients screened for eligibility;
- The number of eligible patients and a comparison with the numbers in the cohort data set. A full trial will be considered feasible if 50% of the patients identified in the cohort study are eligible for inclusion in the trial. Using the lower end of a 95% confidence interval, this gives a minimum of 44% required to be eligible;
- Number of ineligible patients and frequency of predetermined reasons for ineligibility (lack of mental capacity to consent, not living independently, living outside the study area, age, did not have surgery for their hip fracture, living within the study area but outside the area where the intervention could be delivered, other);
- The number of eligible patients recruited (and so by implication were willing to be randomised) will be expressed as a percentage of the numbers in the cohort data set and as a percentage of the numbers identified as eligible in the feasibility study. The full trial will be considered feasible if the expected 25% of eligible patients are recruited to the trial, using the 95% confidence interval this gives a minimum of 18% recruitment;
- The number of eligible patients not recruited and the frequencies and reasons for this (e.g. burden, did not want to be in a research study);
- The number who withdraw after baseline assessment and randomisation and the reasons for withdrawal; and
- The number who completed the various outcome measurements at baseline and at 3-month follow-up. The researchers who administer the outcome measures will record the reasons for any non-completion. The retention rate for the full trial to be feasible is 75%, again using a 95% confidence interval, this gives a minimum of 63% retention for the full RCT to be plausible.

The following demographics descriptors will be presented overall and per randomised group:

- Date of birth (age)
- Gender
- Type of fracture
- Marital status and living arrangements
- Place of residence prior to admission
- Place of discharge from acute or community hospital
- Abbreviated mental test score (AMTS)

All outcome measures will be presented descriptively at all of the time points that they have been collected.

An exploratory correlation analysis will be performed. Correlations will be calculated using Pearson’s product moment correlation coefficient for specific pairs of variables as outlined below. All of the correlations will be completed at both baseline and 3-month follow-up.

- Barthel index versus 8-ft get-up-and-go test.

This is exploring the relationship between the patient’s current functional status and the physical function test used to assess their agility and dynamic balance.

- Barthel index versus general self-efficacy scale/falls efficacy scale-international/self-efficacy for exercise scale.

There are three different self-efficacy measures that are being used within the feasibility stage of this study. All three of these will be correlated with the primary outcome measure to evaluate any differences between them and assist in the decision as to which measure to take forward to a full trial. The correlation is aiming to compare the patient’s self-efficacy with their current functional status to see if a link is present.

Preliminary exploratory analysis of the primary outcome measure (Barthel index) will be completed to find estimates of the means, standard deviations and confidence intervals for both of the treatment arms. These values will also allow the sample size calculation for the future RCT to be calculated. An exploration of any potential differences between the two groups in relation to the Barthel index will be completed using a *t*-test. The effect size and confidence intervals will also be calculated to inform a sample size calculation for a future definitive RCT. It is envisaged that a more complex analysis would be required to elicit an accurate description of the group differences; however, this would only be possible with a larger sample size.

The secondary outcome measures, which are the remaining outcomes at participant level, will follow the same procedure as for the primary outcome measure detailed above, as will the analysis for the only measure taken in relation to the carers (caregiver strain index). The results and appropriateness of the outcomes will be evaluated for continuation for a future RCT.

#### Economic analysis

The enhanced rehabilitation programme will be fully costed using unit costs from a public sector multiagency perspective. Unit costs will be obtained from national sources of reference costs [41,42] and applied to information received from pilot questionnaires, namely salary band of therapists, time spent with the patient conducting rehabilitation, costs of travel and costs of any additional equipment. Costs of health and social care services used by the participants will also be costed using national sources of reference costs. The costs of service use and the cost of the intervention will be added together for use in a cost-effectiveness analysis.

The EQ-5D (3L) will be used to calculate QALYs over the 3-month study period, using area under the curve method [56,57]. An exploratory cost-utility analysis will be conducted to calculate a cost per QALY of the enhanced rehabilitation intervention. This cost per QALY generated will be compared to the NICE threshold range of £20,000 to £30,000 per QALY [58]. If a treatment/intervention costs more than £20,000 to £30,000 per QALY, then it would not be considered cost effective.

The ICEpop CAPability measure for Older people (ICECAP-O) [59] will be used to calculate a capability index score. An exploratory cost-effectiveness analysis will be conducted to calculate a cost per unit change in ICECAP-O score. We will compare the use of these two approaches, cost-utility analysis using QALYs and cost-effectiveness analysis using ICECAP-O, as a capability measure. We will explore the extent to which they can help guide commissioning decisions following a full trial and full economic evaluation.

The FEMuR feasibility study includes a discrete choice experiment [43,44] to look at the participant’s preferences for the ‘process’ of exercise programmes. The discrete choice experiment will assess which attributes are important to participants and will be administered at the 3-month follow-up. Based upon the data received from the feasibility study, we will assess whether the use of a discrete choice experiment is possible from a full scale RCT of the FEMuR multidisciplinary rehabilitation.

Finally, we will scope out the potential of conducting a social return on investment (SROI) analysis based on the data obtained from the feasibility study to inform whether a full SROI analysis is possible from a full-scale RCT of the FEMuR multidisciplinary rehabilitation intervention [60].

#### Triangulation study analysis

We will compare data collected directly from the participants with that obtained from routinely collected data recorded on computerised patient records. There are discrete variables such as number of GP visits, number of hospital visits etc. as well as continuous variables such as medication and associated costs that will be compared for consistency. This comparison will be completed using the intraclass correlation coefficient (ICC) with a value of above 0.7 showing good consistency between the two data sources.

### Focus groups to assess the intervention

The acceptability and feasibility of the different components of this new intervention, including its delivery and the acceptability of being in a randomised study, will be assessed in six focus groups. Three focus groups (*n* = 6 to 8) will be carried out with members of the multidisciplinary rehabilitation teams. Focus groups will be based in each of the three main hospital sites across BCUHB. We will purposively sample a multidisciplinary group of staff who have been involved with delivering the intervention or come into contact with patients involved in the intervention. These staff will include physiotherapists, occupational therapists and their assistant staff and may also include orthogeriatricians, nurses and social workers based in community teams and on the hospital wards. In addition, three focus groups of hip fracture patients and their carers (*n* = 6 to 8) who participated in the enhanced rehabilitation programme intervention will be convened.

#### Patient focus group participants

All patient participants with capacity and their carers will be asked when initially consenting whether they also agree to be invited to a focus group later in their recovery. All those who agree, and who were randomised to the intervention group, will be invited to participate in focus groups. We aim to recruit approximately eight participants to each of the focus groups and should more than eight of those invited wish to attend, we will purposively select [61] participants to cover a range of experiences of rehabilitation. We will ask patients if they would prefer to participate in the focus group through English or Welsh. If enough participants request it, we will run a completely Welsh group. Otherwise, we will run one or more bilingual groups using simultaneous translation to facilitate patient language choice. We will offer patients who move into residential care the alternative of having a telephone or face to face interview. They will be asked to reply within a week to indicate whether they would be willing to take part or not. Researchers will then contact them with details of date, time and location of the focus group being held nearest to their place of residence. Patients and carers who reply to the invitation to the focus group interviews will be contacted by a member of the study team by phone who will explain what will be involved and will go through the information sheet giving them an opportunity to ask questions (similar to Additional file 2). If they still wish to take part in the focus group, they will be given information about when and where the most convenient one for them is taking place.

At the focus group, participants will be given the opportunity to ask questions before being asked to sign the consent form by a researcher. They will be asked to sign two copies of the consent form: one copy for themselves and one for the research team’s records (similar to Additional file 3).

Our experience of running focus groups in phase I of the study was that it is difficult for many patients to attend, particularly those who move to residential care settings. We will therefore offer those individuals an alternative, such as a face-to-face or telephone interview with a researcher.

#### Staff focus group participants

Managers of therapy services will be approached by research staff to help identify staff (e.g. physiotherapists, occupational therapists, nurses and social work staff) who have worked with patients in the intervention arms. Identified staff will be approached by e-mail, phone or letter by study researchers to ask if they would be willing to take part in a focus group and if they know of any other colleagues with experience of the intervention that the researcher could approach. Participants and contacts from the phase I focus groups will be approached to find out whether they have experience of the intervention and whether they would be willing to take part or know of colleagues who might be interested. Staff focus groups will be run in English, but we will check they agree to this and make other arrangements if requested.

Researchers will check that staff participants have received and understood the PIS and will give them opportunity to ask questions about the study at the beginning of the focus group.

#### Focus group procedures

We will have six to eight participants in each focus group. All participants will give written consent to participate and will agree that their comments can be recorded, transcribed and anonymised for analysis. The discussions in the focus groups will be semi-structured and run by a moderator and co-moderator, using a topic guide. The topic guide will be modified in an iterative fashion after each focus group. The topic guide will contain open-ended questions regarding hip fracture patients and their carers’ experiences, perceptions and beliefs about the new rehabilitation programme. Topics covered will be informed by the aims and objectives of the study and the intervention logic model. The discussion will be digitally recorded.

#### Qualitative data analysis

Transcripts of the focus groups will be analysed using the framework approach to thematic analysis [62]. A framework will be developed based on the programme theories, logic model and the questions of feasibility and acceptability this study is designed to answer. The transcripts will be coded and grouped together into categories by the researchers who will have conducted the focus groups. These researchers will discuss, compare and name the categories which will be used to populate and refine the framework. Data that does not fit within the existing framework will be used to develop new sections of the framework that will be agreed amongst the researchers. The framework will be used to develop themes that contribute to answering the study’s feasibility and acceptability questions. Transcript coding and categorising, development of the framework and themes will be reviewed and discussed by the researcher leading on analysis and other members of the team to ensure a rigorous analysis process. This will include appropriate methods for ensuring the findings are plausible and credible. A member of the research team, who has not been involved in the categorising and populating of the framework, will conduct a check of the framework and themes by reviewing several transcripts and assessing whether the coding, categorising and placement within the framework is plausible and credible.

### Trial management

#### Study management group

A study management group (SMG) consisting of individuals responsible for the day-to-day running of the study has been established and is responsible for overseeing the progress of the study throughout all of its phases and meets regularly every 1 to 2 months. The SMG includes the chief investigator (NHW), study manager (CH), study statistician (ZH), trial unit quality assurance manager and study co-applicants. The group ensures that the protocol is adhered to, takes appropriate action to safeguard participants and ensure the overall quality of the study. The SMG reports to the study steering committee (SSC) and the DMEC.

#### Study steering committee

A SSC meeting is being held every 3 to 6 months in order to provide overall supervision of the study and ensure that the study is conducted to the rigorous standards set out in the guidelines for good clinical practice. The SSC consists of the following members: an independent chair (Dr. Sharon Simpson), other independent members (Dr. Fiona Wood, Dr. John Belcher, Prof George Kernohan, Dr. Tom Welsh), patient representative (Ms. Tricia Best), chief investigator (NHW), study manager (CH), members observing from Bangor University as the sponsoring organisation (BW), and a representative from NISCHR-CRC (Mrs. Jayne Jones). It considers study progress and adherence to the protocol and provides advice to the study team. The SSC will make recommendations to the SMG and report to the sponsor and the funder. Terms of reference for the SSC are available on request from the FEMuR study office.

#### DMEC

Data monitoring and quality assurance is being overseen by the DMEC. The DMEC is independent of the study organisers. It considers study progress, recruitment and retention, patient safety and any new information relevant to the study. The DMEC consists of the following members: an independent chair and statistician (Prof. Chris Robertson) and other independent members who are experts in the field of rehabilitation of older people (Prof. Rowan Harwood, Dr. Neil Artz, Dr. Diane Dixon). Terms of reference for the DMEC are available on request from the FEMuR study office. The DMEC will report to the SSC.

### Ethics and regulatory approvals

NHS research ethics (13/WA/0402) and NHS research and development approvals have been obtained. All trial documentation, including participant information sheets, participant consent forms, template GP letters, and questionnaires have been submitted for approval. To conform to the Data Protection Act and Freedom of Information Act, all data will be anonymised and stored securely. No published material will contain patient identifying information.

#### Direct access to source data/documents

Source data will be the hospital written and electronic medical records and routinely collected data, community electronic and written records, audio recordings and transcripts of the focus group interviews. Access to this data will be through members of NISCHR CRC, BCUHB IT staff and researchers on the team who will have NHS research passports. Trial-related monitoring, audits, Research Ethics Committee reviews and regulatory inspections will be permitted, allowing access to data and documents where required.

#### Quality assurance and quality control

This study will be conducted in line with this study protocol and will follow the principles of good clinical practice outlined by the ICH-GCP [63] and will comply with the EU directive 2001/20/EC [64].

Regular monitoring activities will be put in place based on a study risk assessment and delegated to members of the study team to ensure that collected data adhere to the requirements of the protocol; only authorised persons complete Case Report Forms (CRFs); the potential for missing data is minimised; validation checks are performed on the data (e.g. range and consistency checks); and recruitment rates, withdrawals and losses to follow-up are reviewed overall and by hospital site. Only members of the research team who have completed GCP training and have training in focus groups or are supervised by an experienced team member will conduct or be co-moderator at these groups.

#### Data handling

##### Data capturing method

Quantitative data for the feasibility study will be entered into the MACRO data management programme, which is a web-based system allowing controlled access to data by all centres and stores a full audit trail. Additional health service use data obtained from primary and secondary care records will be recorded electronically on encrypted laptop computers or collected by NHS staff on secure computers and anonymised in an electronic data set that is ready for secure transfer to NWORTH.

Data from the focus group interviews will be digital recordings of the focus group discussion and notes taken during the focus groups by the moderator or co-moderator. At the end of the focus group, the recording will be downloaded on to an encrypted NWORTH laptop and subsequently downloaded and stored on the university server in a folder with access limited to core members of the study team. Transfer of the recording to an approved transcriber and return of the transcript will be done by encrypting the recording and uploading to/downloading from a secure server. Written notes will be taken taking care to not to record personally identifiable data, and they will be stored in locked cabinets in locked rooms in NWORTH accessible to authorised team members only.

#### Coding specifications

The design of the source documentation in MACRO will be documented specifying the design, format, derivation and validations used for each type of question in the coding specification. The data captured will be stored in a database running on servers maintained by Bangor University. Access to the complete database will be limited to the core team members of the project involved in data management, data cleaning, analysis and study management. The physical storage of paper case report forms will be documented within the data management plan. The coding will be conducted in the design set-up phase of the source documentation for MACRO. The code book will be shared along with the data in the data sharing process to allow meaningful interpretation of the data set by other researchers in the project.

#### Data transfer process steps

Data from the focus group notes and transcriptions of the discussion will be transferred to NVIVO [65] or Excel™ software for qualitative analysis. Data from the feasibility study on the MACRO data management programme will be made available for analysis via SPSS 20.0 [66]. Paper copies of case report forms (participant questionnaires) will be stored securely on Bangor University premises during the trial. Photocopies, if needed, will be made before returning any originals to NWORTH. The originals will be returned to NWORTH via recorded delivery/courier for data entry, if necessary, and for archiving at the end of the study. The photocopies held at the site will be destroyed at the end of the trial once all the final data set is closed. Consent forms wherever possible will be stored securely at the NHS sites.

Any consent forms (e.g. focus group consent forms) and paper recorded data stored at Bangor University will be kept in separate locked cabinets.

#### Review of the quantitative data

A periodic review of the data will be performed to ensure accuracy of data entered into the database. The researchers entering the data into online system will randomly check each other’s entries against the paper CRFs to ensure consistency and accuracy, determine if all participant data has been entered and checked for missing values, and identify any obvious problems. A random check of ID, number of entries and out-of-range values will be also performed.

#### Data management

A data management plan has been written which covers processes for auditing, cleaning and monitoring quality. The transcripts of the focus groups will be checked for accuracy of transcript by one of the researchers who attended the focus group and by using the audio recording as necessary. The transcripts will be checked for any identifying data such as names and places and these will be removed or replaced with a description of what sort of information it was so the transcript still makes sense and to ensure anonymity of the participants.

#### Data sharing

Data will be shared with the members of the research group when required. The member may formally request for a specific data set using a data request form which is included in the data management plan. All such requests will need to be approved by the chief investigator (NHW). All quantitative data will be accompanied by a copy of the relevant codebook. The request and the data set provided to which member will be recorded and saved in respective folders named after the member.

#### Data archiving

Data archiving details the storage of the data after the study has ended complete with the relevant audit trail that will allow tracking from raw entered data to the final master data set used for analysis. The storage location of hard copy data will be recorded in the data management plan. At the end of the study original data, analysis data and the data tracking file will be archived with access only to authorised people.

#### Publication policy

##### Dissemination plan

A publication strategy has been developed. We are committed to publishing in a wide range of peer-reviewed journals in multiple disciplines, e.g. rehabilitation medicine, physiotherapy, health psychology, and to ensuring that appropriate recognition is given to all who have worked on the study. We are also committed to making research data accessible for secondary analysis. We will also disseminate the results to the teams that look after patients with proximal femoral fracture in Betsi Cadwaladr University Health Board, in the acute hospitals, the community hospitals and the community rehabilitation teams and at scientific meetings to primary and community care, orthopaedic and rehabilitation audiences.

##### Authorship eligibility

To qualify as an author, the author will have made substantial contributions to the conception and design, or acquisition of data, or analysis and interpretation of data; been involved in drafting the manuscript or revising it critically for important intellectual content; given final approval of the version to be published with each author having participated sufficiently in the work to take public responsibility for appropriate portions of the content; and agreed to be accountable for all aspects of the work in ensuring that questions related to the accuracy or integrity of any part of the work are appropriately investigated and resolved [67].

#### Indemnity

Bangor University has appropriate Clinical Trials Indemnity and Professional Indemnity insurance in place that will cover members of the research team to conduct the research as per protocol. NISCHR CRC staff has NHS contracts and will be responsible to ensure that their work is appropriately insured. NHS and social services staff who work with patients involved in the intervention will not be expected to do anything that is not covered by their contracts and will remain covered by the NHS or social services insurance arrangements.

## Discussion

The enhanced multidisciplinary rehabilitation programme has been developed from programme theories obtained from a realist review of the hip fracture rehabilitation literature, with contributions from a survey of current practice and focus groups of patients, carers and rehabilitation team members. In this randomised feasibility study, we will use mixed methods to examine different aspects of this programme: its acceptability in focus groups; quality of life, functional ability, mental health, potential psychological mediators of outcome and health service use with patient completed outcome measures; objective measures of physical function; patients’ preference for the content of the programme in a discrete choice experiment; routinely collected information to measure health service use and process outcomes; and adverse event reporting. The criteria for judging the feasibility of a future RCT will be an eligibility rate of greater than 50% of those screened (lower bound of a 95% confidence interval (CI) would be 44%), recruitment rate greater than 25% of those eligible (lower bound of a 95% CI would be 18%), retention rate at 3-month follow-up greater than 75% (lower bound of a 95% CI would be 63%).

Phase II studies are primarily intended to test the feasibility and acceptability of the various trial methods prior to a larger definitive phase III randomised controlled trial. However, they also provide an opportunity to compare and contrast different methods such as patient-completed questionnaires with objective measures of physical function; collecting health resource data from routinely collected information with patients’ self-report; different process and outcome measures for self-efficacy, functional ability and quality of life; and different objective measures of physical function.

We have also described how we envisage our complex rehabilitation intervention works in a logic model whose starting point was the programme theories [15], through to short-term and long-term goals, and finally, how these influence an individual’s impairments, activity and participation according to World Health Organisation’s ICF model [24]. Some of the process outcomes to be measured can be used to start to explore this model such as the three measures of self-efficacy and participants’ engagement with the personal goal setting set out in the workbook and goal-setting diary. Rehabilitation therapy self-efficacy has been shown to be associated with functional recovery in a small US cohort study [23] and also from a systematic review in the related field of joint replacement surgery [8]. The logic model will also be used to inform the framework developed for qualitative data analysis. This will add to its evaluation and also potentially provide valuable data on how to refine the model and the intervention for a future definitive RCT.

### Trial sponsor

Bangor University: sponsor’s reference 11/33/03.

Contact: Professor Bob Woods, Schools of Healthcare and Medical Sciences, Ardudwy, Normal Site, Holyhead Road, Bangor, LL57 2AS. Tel: (01248) 383719. Fax: (01248) 382229.

### Ethical approval

Ethical approval was obtained from the North Wales West Research Ethics Committee (REC reference 13/WA/0402).

## Trial status

The randomised feasibility study has research ethics and R&D approval and has started to recruit participants.

## Additional files

Additional file 1:
**Participant information sheet: patient.**

Additional file 2:
**Fracture in the Elderly Multidisciplinary Rehabilitation (FEMuR) study.**

Additional file 3:
**Adverse event reporting.**

## Abbreviations

(S)AE: (Serious) Adverse events
ADL: Activities of daily living
AMTS: Abbreviated Mental Test Score
BCUHB: Betsi Cadwaladr University Health Board
CI: Confidence interval
CRC: Clinical research collaboration
CRF: Case report form
CSI: Carer strain index
CSRI: Client service receipt inventory
DMEC: Data Monitoring and Ethics Committee
EQ-5D (3 L): EuroQol-5 dimensions (3 levels)
EU: European Union
FES-I: Falls Efficacy Scale - International
GP: General practitioner
GSES: General Self-Efficacy Scale
HADS: Hospital Anxiety and Depression Scale
ICECAP-O: ICEpop CAPability measure for older people
ICF: International Classification of Functioning, Disability and Health
ICH-GCP: International Conference on Harmonisation of Technical Requirements for Registration of Pharmaceuticals for Human Use. Guideline for Good Clinical Practice
IT: Information technology
MRC: Medical Research Council
NEADL: Nottingham Extended Activities of Daily Living
NHS: National Health Service
NICE: National Institute of Health and Clinical Excellence
NISCHR: National Institute of Social Care and Health Research
NWORTH: North Wales Organisation for Randomised Trials in Health
PIS: Patient information sheet
POVA: Protection of vulnerable adults
QALY: Quality adjusted life year
RCT: Randomised clinical trial
SMG: Study management group
SSC: Study steering committee
VAS: Visual Analogue Scale
VAS-FoF: Visual Analogue Scale - Fear of Falling
