# Supplementary material for: Fracture in the Elderly Multidisciplinary Rehabilitation (FEMuR): study protocol for a phase II randomised feasibility study of a multidisciplinary rehabilitation package following hip fracture [ISRCTN22464643]
Source: Pilot Feasibility Stud. 2015 Apr 7;1:13. doi: 10.1186/s40814-015-0008-0 (PMC5154127; doi:10.1186/s40814-015-0008-0)
Supplement: Additional file 1: — Participant information sheet: patient. [file 40814_2015_8_MOESM1_ESM.docx]

**Appendix 1 Participant information sheet**

**Participant Information Sheet: Patient**

**Fracture in the Elderly Multidisciplinary Rehabilitation (FEMuR) Study Phase 2**

You have been invited to take part in a research study. Before you decide whether to take part, it is important for you to understand why the study is being undertaken and what it involves. Please take time to read the following information carefully and discuss it with others if you wish. If there is anything that is not clear or if you would like more information you can contact us, details on page 4 below.

**What is the FEMuR study about?**

Hip fracture is a common health problem in old age, especially for people who have other health problems, or are frail. Many people who could look after themselves before their fracture lose their independence afterwards. To have the best chance of making a good recovery, it is recommended that patients should be assessed by a specialist doctor and start on a programme of rehabilitation soon after surgery that continues in the community after they leave hospital. Occupational Therapists and Physiotherapists plan and oversee progress of patients on rehabilitation programmes. We have designed a new way of providing a rehabilitation programme and want to assess if this programme is acceptable to patients, carers and clinicians. We want to compare this programme with the one that is currently available to see which method is the most effective and gives the best value for money.

We also want to see if we can reduce the number of questions patients have to complete when taking part in healthcare research. We will check to see if the same quality information is available from the electronic records held in hospitals and GP practices in the Betsi Cadwalader University Health Board. In this study we will ask patients the questions and then compare their answers with the information that can be collected from the electronic records.

**Why have I been chosen to take part?**

You have been asked to take part because you recently had surgery for a fractured hip in a North Wales hospital.

**Do I have to take part?**

No, participation in this project is completely voluntary. You have been given this information sheet to keep. If you decide to take part you will be asked to sign a consent form and be given a copy of the form. If you decide to take part you are still free to withdraw at any time and without giving a reason. A decision to withdraw at any time, or a decision not to take part, will not affect the usual care that you receive.

**What will I be asked to do if I decide to take part?**

We need to establish whether or not the new rehabilitation programme is better. To do this we need to compare it with the standard programme of rehabilitation that is currently offered in North Wales. The fairest way of doing this is to select people by chance; everyone agreeing to take part will have a 50:50 chance of being offered a place on the new rehabilitation programme or the standard programme. Selection is made by computer, which will not use any information that would identify you. This kind of study is known as a ‘randomised feasibility trial’.

If you decide to take part you will be asked to complete a questionnaire and measure your hand grip strength before you are discharged from the acute hospital. After three months we will ask you to complete a follow-up questionnaire about your health and fitness and a set of physical tests to assess your physical function following the rehabilitation programme. A researcher will contact you to make an appointment at a time that suits you to do this at your place of residence.

We will also ask you for permission to extract data about the services you used from the records that Betsi Cadwalader University Health Board keeps. This will include notes made by the hospital and your GP.

Around the time of your follow up visit, if you are in the group that is offered the new rehabilitation programme we will invite you and anyone who cares for you to take part in a group interview with other patients who have followed the same programme. The purpose of the group discussion is to find out your experiences of the new programme. You don’t need to decide immediately if you want to take part in the group discussion. You can decide when you receive the invitation to take part.

**What happens if my ability to make my own decisions changes whilst I am taking part in the study?**

Your ability to understand new information and make decisions about it is called your mental capacity. If you lose this ability because of illness or disability then you will not be expected to continue to take part in the trial. Researchers will use the data that has already been collected but they will not collect any new information about you or your care from that point onwards. A change in your mental capacity and withdrawal from the trial will not affect your normal NHS care, this will continue as usual.

**Will my taking part in this study be kept confidential?**

All information collected in this study will be kept strictly confidential. Only members of the research team will have access to it. No names or details identifying specific individuals will be included in study reports.

##### What are the possible disadvantages and risks of taking part?

##### We do not foresee any disadvantages or risks to you taking part in the study, although the assessments will, of course, take up some extra time.

##### What are the possible benefits of taking part?

##### Although there may be no direct benefits to you from taking part in this study, the information you give us will be used to assess the new rehabilitation programme for older people with hip fractures. People who take part in studies like these often report that they enjoyed it and benefited just by being involved and that they were pleased that it could help people in future.

**What happens if something goes wrong?**

We do not foresee any circumstance where you will come to harm. So if you are harmed by taking part in this study, there are no special compensation arrangements. However, if you are harmed due to someone’s negligence, then you may have grounds for a legal action, but you may have to pay for your legal costs.

If you are unhappy or dissatisfied about any aspect of your participation, we would ask you to tell us about this in the first instance, so that we can try to resolve any concerns and find a solution. Regardless of this, if you wish to make a complaint about any aspect of the way you have been approached or treated during the course of this study, the normal National Health Service complaints procedures should be available to you.

**What will happen to the results of the study?**

The results will be written up for publication in a medical journal. The details will be submitted for presentation at local and national orthopaedic meetings so that the findings can be used by doctors and therapists in the future. You will also be asked if you would like a copy or a summary of the study findings.

**Who is organising and funding the research?**

We have obtained a grant from the National Institute for Health Research. The Chief Investigator is Dr Nefyn Williams from Bangor University and his team includes researchers, doctors and other health professionals from Bangor University and the Betsi Cadwaladr University Health Board.

**Who has reviewed the study?**

The study has been reviewed and approved by the Research Ethics Committee for North Wales.

**What do I do now?**

A member of the research team will contact you in the next few days. If you agree to take part in the study you will be asked to sign three copies of the accompanying consent form. One copy of the consent form and this information sheet will be for you to keep. The second copy of the consent form will be placed in your hospital file and the third copy will be retained by the research team.

We very much appreciate the time that you have taken in reading through the details of the study.

**Who can I contact for further information?**

For more information about this research, please contact:

**Dr Claire Hawkes**

Trial Manager, FEMuR

North Wales Organisation for Randomised Trials in Health (NWORTH)

Y Wern, Normal Site

Bangor University

Gwynedd, LL57 2PZ

Telephone: 01248 382224

E-mail: [c.hawkes@bangor.ac.uk](mailto:c.hawkes@bangor.ac.uk)

**What should I do if I have any concerns about the study?**

If you have any concerns about the study, please contact the Chief Investigator in the first instance. If you continue to have concerns you can contact the Concerns Team at Betsi Cadwaladr University Health Board. Their names and contact details are as follows:

**Concerns Team**

Betsi Cadwaladr University Health Board

Ysbyty Gwynedd

Penrhosgarnedd

Bangor

Gwynedd

LL57 2PW

Telephone: 01248 384384

**E-mail:** [ConcernsTeam.bcu@wales.nhs.uk](mailto:ConcernsTeam.bcu@wales.nhs.uk)

**Dr Nefyn Williams**

Chief Investigator, FEMuR

North Wales Organisation for Randomised Trials in Health (NWORTH)

Y Wern, Normal Site

Bangor University

Gwynedd, LL57 2PZ

Telephone: 01248 388095

E-mail: [nefyn.williams@bangor.ac.uk](mailto:nefyn.williams@bangor.ac.uk)
