# Supplementary material for: Fracture in the Elderly Multidisciplinary Rehabilitation (FEMuR): study protocol for a phase II randomised feasibility study of a multidisciplinary rehabilitation package following hip fracture [ISRCTN22464643]
Source: Pilot Feasibility Stud. 2015 Apr 7;1:13. doi: 10.1186/s40814-015-0008-0 (PMC5154127; doi:10.1186/s40814-015-0008-0)
Supplement: Additional file 2: — Fracture in the Elderly Multidisciplinary Rehabilitation (FEMuR) study. [file 40814_2015_8_MOESM2_ESM.docx]

**Appendix 2 Participant informed consent form**

**Fracture in the Elderly Multidisciplinary Rehabilitation (FEMuR) Study**

Participant identification number:

PARTICIPANT CONSENT FORM

1. I confirm that I have read and understand the participant information sheet dated 26/03/2014 (version 3) for this study and I have had the opportunity to ask questions about the study
2. I understand that my participation is voluntary and that I am free to withdraw at any time, without giving any reason. I understand that if I withdraw this will not affect my healthcare or legal rights in any way. If I withdraw from the study the researchers will use the information I have provided up to that point, unless I indicate that I do not want them to.
3. I understand that I will be assigned to the intervention or control group at random and that if I am in the control group I will receive usual care.
4. I understand that the information I give to the researchers will only be used for the purposes of research, and that personal details will be treated in the strictest confidence.
5. I understand sections of my medical notes will be accessed and used by individuals involved in the trial or from regulatory authorities where it is relevant to my taking part in the research. I give my permission for these individuals to have access to my NHS records, including hospital notes, GP notes and rehabilitation therapy notes, and for details from these records to be linked to the trial data to provide additional information to support the research.
6. I understand that if I lose my mental capacity I will be withdrawn from the study and the researchers will use the information I have provided up to that point, but will not collect any more information about me or my care.
7. I understand that I will be asked questions about my health and mood at the beginning and end of the study and that a physiotherapist will test my physical function at the end of the study.
8. I understand that if the researchers have any serious concerns about my health, safety or well-being, they have a duty to inform my GP or another appropriate professional
9. I agree to my GP and my hospital consultant being informed that I am taking part in this study.

1. I agree to be contacted about attending a focus group in the future and understand that there is no obligation for me to attend this focus group, even if I take part in the study.
2. I agree to take part in this study.

Name of participant Date Signature

Researcher taking consent Date Signature

**One copy for participant; one copy for the patient’s hospital file, one copy for researcher.**

**Should you have any queries concerning this research, please contact:**

Dr Nefyn Williams
Chief Investigator, FEMuR
North Wales Organisation for Randomised Trials in Health (NWORTH)
Y Wern
Normal Site
Bangor University
Gwynedd
LL57 2PZ
Tel: 01248 388095
Email: nefyn.williams@bangor.ac.uk
