# Supplementary material for: Fracture in the Elderly Multidisciplinary Rehabilitation (FEMuR): study protocol for a phase II randomised feasibility study of a multidisciplinary rehabilitation package following hip fracture [ISRCTN22464643]
Source: Pilot Feasibility Stud. 2015 Apr 7;1:13. doi: 10.1186/s40814-015-0008-0 (PMC5154127; doi:10.1186/s40814-015-0008-0)
Supplement: Additional file 3: — Adverse event reporting. [file 40814_2015_8_MOESM3_ESM.docx]

**Appendix 3 Adverse event reporting**

**Instructions**

1. Upon becoming aware of an adverse event (AE) involving a participant or carer, record it on the attached form in part A. There is no need for you to determine whether it is “serious” as this will be done by the chief investigator (CI) of the study once it has been reported.

An AE is an untoward occurrence experienced by either a participant or carer which:

1. exacerbates a pre-existing illness; (e.g. something that causes an acute asthma attack necessitating a change in medication use or visit to the hospital for someone who has asthma).
2. increases in frequency or intensity a pre-existing episodic condition (e.g. an increased frequency of angina episodes necessitating taking medication or other health service intervention – these may or may not be directly as a result of the study (e.g.directly related might be: following practice of physiotherapy extercises);
3. is a condition detected after the start of the study (even though it may have been present prior to the start of the study) (e.g. a new diagnosis of Parkinson’s disease after recruitment to the study)
4. is a continuous persistent disease or symptoms present at baseline that worsen during the study (e.g. periferal vascular disease present at baseline with intermittent claudication symptoms provoked by shorter distances walked);
5. results in a fall or repeat fracture;
6. requires hospitalisation (e.g. a myocardial infarction that happended at home resulting in a hopsitialisation)or prolongs existing hospitalisation (whilst recovering from hip fracture surgery the patient constracts an infection requiring inpatient IV medication);
7. results in persistent or significant disability or incapacity (e.g. a stroke resulting in hemiplegia that doesn’t resolve completly as might happen with a TIA);
8. is otherwise considered medically significant and based upon appropriate medical judgement, may jeopardise the participant and require medical or suirgical intervention;
9. is life-threatening;
10. results in death.

In addition, any cases where action has been taken for the protection of vunerable adults (dealing with suspected abuse or neglect of participants) should be reported to Bangor using this procedure.

It should be noted that all AE should be reported to Bangor University, even if initially there may be no obvious connection to the trial. In particular:

- All deaths of participants and carers should be reported to Bangor University.
- All incidents of hosptialisation (and prolonging of hospitalisation) for participants and carers should be reported to Bangor (even when the illness or condition being treated has no obvious connection to the trial).

1. If an AE is deemed to have taken place, therapists, clinicians and researchers should complete the attached form part A and forward it to the StudyManager as soon as possible and within 24 hours, where possible, of discovering and AE has taken palce. The contact details are given at the end of part A.
2. The Study Manager will liase with the study CI who will determine whether it is serious or not and whether it is realated to the study or not.
3. The CI will report and SAEs to the DMEC chair and the CI and DMEC Chair will determine whether they are related to the study. If the AE is determined to be both serious and related to the study, the CI will report it to the sponsor and to the academic school (Schools of Medical and Healthcare Sciences, College of Health and Behavioural Science) within 24 hours. They will also be reported to the Research Ethics Committee.

**Flow chart of FEMuR Serious Adverse Event Reporting Procedure**

**Yes**

Is the incident assessed as serious and is it related to the study?

In the FEMuR study a Serious Adverse Event (SAE) is an untoward occurrence, experienced by a participant or carer, which:

- resulted in death;
- Was life-threatening;
- Required hospitalisation or prolongation of existing hospitalisation;
- Was a fall or repeat fracture;
- Results in persistent/significant disability or incapacity;
- Is otherwise considered medically significant by the Chief Investigator.

Or

- Alleged/suspected abuse/neglect

No further action required.

**No**

1. The Chief Investigator (CI) should complete Part C of the SAE Form.
2. Where the SAE is deemed to be related to the FEMuR study, the CI will notify (within 15 days) the following:
3. REC;
4. Study DMEC.
5. The SAE Form should be filed in the Trial Master File (TMF)
6. The CI should complete Part B of the SAE Form electronically, as far as possible.
7. The CI should forward this form to the sponsor, trial manager and the academic school. The trial study manager will keep the form in the investigator’s site file.

Notification of adverse event received from researcher, clinician or GP using part A of the AE form.

**FEMuR Serious Adverse Event Reporting Form**

**PART A (to be completed by Researcher, Therapist or GP)**

**A1.** Centre Name: **BCUHB**

Completed by: **_______________________________________________**

|  |  |  |  |  |  |  |  |
| --- | --- | --- | --- | --- | --- | --- | --- |

**A2.** Date form completed:

d d m m y y y y

|  |  |  |  |  |  |
| --- | --- | --- | --- | --- | --- |

**A3.** Participant Identity Number:

**A4.** How did you become aware of this incident?

**A5.** Was this AE suffered by the participant or carer? *Place an* ***“x”*** *in* ***one*** *box only.*

Participant

Carer

**A6.** Are you reporting a death? Place an “x” in **one** box only.

Yes **Please proceed to Question A8**

No **Please proceed to Question A7**

**A7.** Please categorise this event, by placing an “x” in **all** appropriate boxes.

Life threatening

Hospitalisation or prolongation of existing hospitalisation

Persistent or significant disability or incapacity

Otherwise considered medically significant

|  |  |  |  |  |  |  |  |
| --- | --- | --- | --- | --- | --- | --- | --- |

**A8.** Date of SAE

d d m m y y y y

**A9.** Location of SAE **_____________________________________________________________**

Describe the

circumstances of the

event. Is there any

**A10.** evidence that

participation in the

trial may have been

a contributing factor?

(attach further sheet

if necessary)

After signing, please send by post, or email to the address below and retain a copy for your records. You can also contact the Study Manager by phone during office hours.

Dr Claire Hawkes

Study Manager for FEMuR

Y Wern

The Normal Site

Holyhead Road

Bangor

Gwynedd

LL57 2PZ

c.hawkes@bangor.ac.uk

Tel: 01248 383243

**PART B (to be completed by Chief Investigator)**

**B1.** In your opinion, is this reported AE assessed as serious according to the FEMuR protocol?

Yes

No

**B2.** In your opinion, did this AE arise as a result of the participant’s or carer’s involvement in the Femur Study? Place an “x” in **one** box only.

Yes

No

**B3.** Please add any comments

regarding the SAE

Please print and sign a copy of this form and return to Dr Claire Hawkes for the investigator’s Site File.

**PART C (to be completed by Chief Investigator)**

**C1.** Action taken

**C2.** Name of CI Dr Nefyn Williams

**C3.** Signature of CI

|  |  |  |  |  |  |  |  |
| --- | --- | --- | --- | --- | --- | --- | --- |

**C4.** Date of signature

d d m m y y y y
